# Case of Nasopharyngeal Carcinoma Presenting With Rare Combination of Multiple Cranial Nerve Palsies

**DOI:** 10.7759/cureus.20357

**Published:** 2021-12-12

**Authors:** Alexa J Denton, Arjun Khunger, Andres Reyes-Corcho

**Affiliations:** 1 Medicine, Florida International University, Herbert Wertheim College of Medicine, Miami, USA; 2 Internal Medicine, Memorial Hospital West, Pembroke Pines, USA

**Keywords:** cranial nerve palsies, cranial nerve, percutaneous endoscopic gastronomy, dysphagia, bell’s palsy, nasopharyngeal carcinoma

## Abstract

Cranial nerve palsies are commonly known comorbidities associated with nasopharyngeal carcinoma, occurring in nearly 20% of cases. These palsies occur in isolation or in common groupings, depending on the anterior or posterior cranial vault extension of the lesion. Cranial nerve VII palsy is relatively rare, with an incidence of less than 1%. As a poor marker of prognosis, cranial nerve involvement may lead to significant morbidity amongst patients with nasopharyngeal carcinoma.

We report a case of a 73-year-old male diagnosed with nasopharyngeal carcinoma with extension into the skull base who presented with both anterior and posterior cranial nerve involvement throughout the course of his disease. With lesions in cranial nerves III, V, VI, VII, IX, and XII, this patient experienced a sequence of right-sided facial paralysis, facial pain, inability to abduct his right eye, rightward tongue deviation, tinnitus, hearing loss, decreased extraocular eye movement superiorly, and dysphagia which subsequently worsened with chemotherapy and radiation. Most notably, he presented with a right-sided cranial nerve VII palsy, not commonly reported in the literature.

## Introduction

Nasopharyngeal carcinoma (NPC) is a historically rare malignancy with an incidence of less than one per 100,000 person-years and is most predominantly found in males indigenous to Southeast Asian countries [1]. Due to the anatomic location of the nasopharynx and its direct connection to adjacent head and neck structures, patients with NPC may present with a wide variety of symptoms including isolated neck masses, nasal congestion, epistaxis, visual and/or auditory deficits, cephalalgia, or cranial nerve (CN) dysfunction. Some of these symptoms, in isolation or even in combination, may not initially draw suspicion for a diagnosis of NPC due to their associations with other head and neck conditions and difficulty in accessing this region on the physical exam [2].

Usually, CN involvement due to NPC presents in either single palsies or in grouped CN syndromes, depending on the local spread of the tumor [2]. Cranial nerve VII is not commonly reported as either an isolated palsy or part of a syndrome. These palsies can be an initial presentation, a later presentation of known disease course as a prognostic indicator, or post-radiation and chemotherapy side effects [2]. We report an unusual case of NPC in a patient with a known history of resolved Bell’s palsy initially presenting with a recurring episode of CN VII dysfunction and facial pain. Subsequently, the clinical course was complicated with the progression of multiple upper and lower CN deficits prior to initiation of treatment.

## Case presentation

A 73-year-old Hispanic man with a medical history of hypertension, and type 2 diabetes mellitus presented to the emergency department (ED) with a chief complaint of right-sided facial pain, facial droop, and ptosis. Eight years prior, the patient had an episode of right-sided Bell’s palsy characterized by incomplete right-sided facial paralysis, which resolved spontaneously without any complications. The right facial and eyelid droop began two weeks prior to presenting to the ED while the severe, sharp, constant facial pain began one day prior to presentation prompting him to come to the ED. No other neurological findings were seen on physical exam and all laboratory results were within normal limits. The patient was discharged from the ED with a prescription of Percocet and advised to follow up with an outpatient neurologist for further work-up.

**Figure 1:**
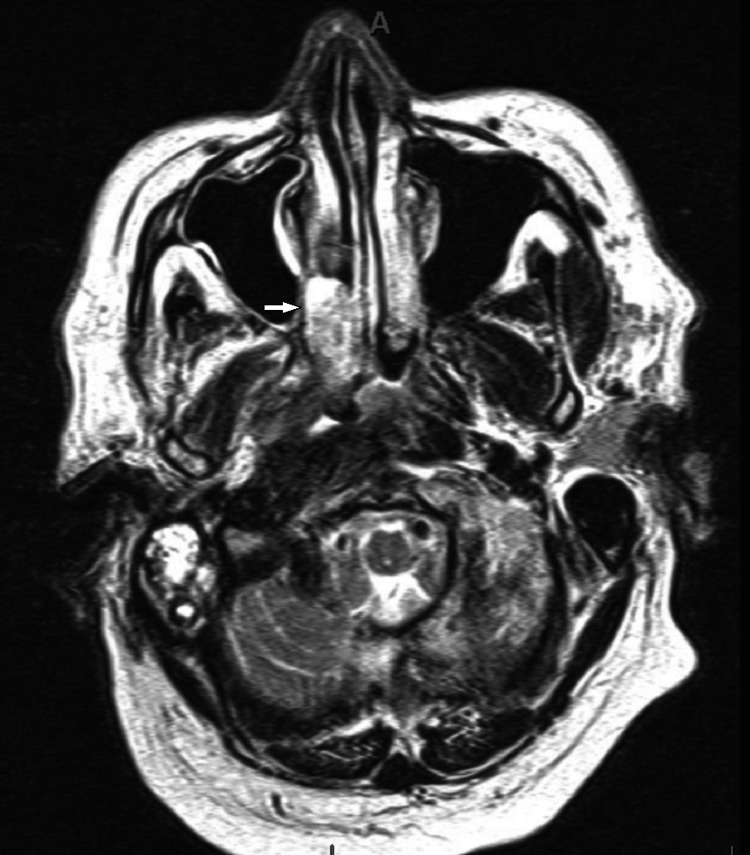
MRI of nasopharyngeal carcinoma Head magnetic resonance imaging of the neoplasm (arrow) shows an ill-defined infiltrating mass in the posterior right aspect of the nasopharynx extending into the skull base.

One month later, the patient underwent a gadolinium-enhanced brain magnetic resonance imaging (MRI) which revealed an ill-defined infiltrating enhancing mass in the posterior right aspect of the nasopharynx with extension and infiltration of the skull base and was highly suspicious for a neoplasm (Figure 1). The patient followed up with an outpatient otolaryngologist and underwent a computed tomography (CT) sinus, CT sinus with contrast, and positron emission tomography (PET) which confirmed the neoplasm. A flexible laryngoscopy with biopsy was also performed which revealed the known mass in the right posterior nasal cavity, which extended into the nasopharyngeal space and nearly occluded the right nasal cavity. There was also evidence of crusting in both inferior turbinates, inflammation noted in the right middle meatus, and lesion extension in the right eustachian tube orifice. There was no extension of the mass into or beyond the oropharynx. At this time, the patient continued to have right-sided facial pain and paralysis with newly associated symptoms of right-sided hearing loss, right ear pain, diplopia in the right eye, epistaxis, sinus pressure, and voice change. The patient denied any dysphagia or odynophagia. On physical exam, tongue deviation to the right and the inability to abduct the right eye when examining extraocular eye movements (EOM) were appreciated. These findings suggest the involvement of multiple CNs including CN V, CN VI, CN VII, and CN XII.

Overall, pathology and imaging reports revealed the diagnosis of nasopharyngeal squamous cell carcinoma tumor, nodes, and metastases (TNM) staging: T3 N2 M0 Stage III with an extension of the mass to the adjacent structures both anteriorly and posteriorly. Additionally, there was evidence of both mass infiltration into the nasal cavity and locoregional metastasis into the neck lymph nodes bilaterally. The tumor was also found in the skull base region with erosion and interarticular extension into sphenoid sinuses bilaterally. The outpatient otolaryngologist determined that this patient was not a candidate for surgical resection and would follow up with oncology for chemotherapy and radiation. Further explanation of surgical versus medical treatment for NPC will be outlined in the discussion.

One month following definitive diagnosis, the patient presented to the ED prior to initiating chemotherapy and radiation treatment with new onset of severe headaches, tinnitus, facial paresthesias, dysphagia, nausea, and vomiting. He continued to present with the symptoms on initial presentation of right-sided facial pain, droop, and ptosis. He also reported losing 40 pounds in three months and inability to swallow solid foods. With suspected involvement of CN IX and new-onset headaches, CT imaging was performed which reported stability of the nasopharyngeal mass without any acute intracranial hemorrhages or midline shifts. He was given Tylenol for pain and advised to report to outpatient oncology to initiate treatment.

Five days following his first chemotherapy and radiation session, the patient presented to the ED with severe fatigue, dehydration, worsening dysphagia, and odynophagia. The patient was only consuming liquid meal replacements three times daily due to dysphagia for the past three months. Physical exam revealed consistent CN findings with documentation of decreased upward gaze when examining EOMs, suggestive of CN III involvement. Laboratory studies showed signs of dehydration secondary to poor oral intake including hyponatremia (131 mmol/L), hypokalemia (3.3 mmol/L), and hypochloremia (95 mmol/L), acute kidney injury (AKI) with significant elevation in blood urea nitrogen (BUN), and creatinine from baseline values within normal limits to 34 mg/dL and 1.99 mg/dL, respectively, The patient was initiated on intravenous (IV) fluids and gastroenterology was consulted for percutaneous endoscopic gastrostomy (PEG) tube placement due to severe dysphagia. Following resolution of electrolyte imbalance and AKI, the PEG tube was surgically inserted and tube feeding with Neutron was started. The patient reported significant improvement in fatigue, tolerated bolus feeding without nausea, vomiting, or abdominal pain, and was advised to follow up with an outpatient oncologist following discharge. 

## Discussion

This is a case of a T3 N2 M0 staged squamous cell NPC with significant expansion into the neighboring structures of the nasopharynx and cervical lymph nodes. Generally, surgical resection does not play a large role in the initial treatment for NPC regardless of the staging, with chemoradiotherapy serving as the gold standard [3]. This is because the anatomical region is a difficult target to navigate for full tumor resection with clear margins [4]. Additionally, these neoplasms are historically responsive to a combination of radiation with or without chemotherapy, depending on the staging [4]. Chemotherapy will be added to the regimen when a tumor is classified as at least stage II [5]. The role that surgery plays in these cases is reserved for when the neoplasm reoccurs the following chemoradiotherapy, leading to the potential use of salvage surgery with neck node dissection for curative intent [3,5]. Not all patients are candidates for these procedures, as there are high morbidity and complication rates, depending on the type of head and neck cancer [5]. It is also important to mention the common effects that medial management can lead to, as it relates to our case. Au et al. [6] conducted a retrospective review study of 3328 patients with NPC who underwent intensity-modulated radiotherapy. This revealed that CN palsies were evident in 5.1% of patients, severe hearing loss was present in 7.1% of patients, and dysphagia requiring long-term feeding was evident in 3% of patients at a median follow-up of 80 months [6]. With this study elucidating the side effects of already known NPC, we will continue to discuss CN involvement as an initial clinical presentation and following the initiation of treatment. 

CN palsies are found in approximately 20% of NPC cases and may clue physicians into working up the possible diagnosis of locally invasive NPC [2]. These nerve dysfunctions can be seen as a single palsy or in syndromes that involve multiple CNs depending on the direction of intracranial spread [7]. Common combinations of these palsies also depend on the direction in the cranial vault of tumor extension. If the tumor has invaded anteriorly towards the cavernous sinus and middle cerebral fossa regions, there can be pronounced visual, oculomotor, and facial sensation disturbances due to possible impaction of CN II, III, IV, V, or VI. In this grouping, CN V and VI are the most commonly affected, with symptoms of facial pain and decreased eye abduction on presentation, respectively [8]. Posterior invasion into the skull base may lead to vocal cord dysfunction, dysphagia, tongue deviation, and trapezius and sternocleidomastoid muscle weakness via extension into the nerves that traverse the jugular foramen and hypoglossal canal. These include CN IX, X, XI, XII [7]. 

In this case, the patient presented with CN V dysfunction as evident by the trigeminal neuralgia experienced throughout all clinical presentations. The patient’s pain traversed all three branches of CN V on physical exam, consistent with probable tumor extension into the superior orbital fissure, foramen rotundum, and foramen ovale. The involvement of CNs III and VI were also evident in the patient, completing a clinical picture similar to superior orbital fissure syndrome that has been described in the literature [2]. This has been reported with clinical manifestations such as total ophthalmoplegia of CN III EOM, eye abduction deficits, trigeminal neuralgia or paresthesias in the ophthalmic nerve distribution, proptosis, and a mydriatic pupil [2]. Our patient only presented with an upward gaze deficit and no evidence of impaired pupillary reflex, possibly suggesting incomplete compression of CN III. The lesion in this case also infiltrated into the posterior aspect of the skull base as corroborated by both imaging and the clinical manifestations of ipsilateral tongue deviation and late presenting dysphagia. Both extensive upper and lower cranial nerve presentations with this specific combination in the same patient due to NPC have not been found to be reported in the literature.

Isolated involvement of CN VII has not been widely reported as an initial clinical manifestation of NPC and may occur in less than 1% of patients with NPC [9]. Three previous case reports with facial nerve palsy due to NPC were reported in the case series by Low [9]. Each patient presented with symptoms of facial paralysis, with one patient presenting with hearing loss and another presenting with tinnitus, all of which were experienced by the patient in our case. The CN VII palsy clinical presentation due to NPC likely varies due to the complex anatomical course the nerve takes while exiting the skull and to innervate its respective structures. This nerve arises from the pons, enters the cerebellopontine angle, and exits the skull via the stylomastoid foramen [10]. CN VII also has a wide range of sensory and motor functions associated with facial movement, taste, and hearing [10]. In this case, CN VII palsy presenting as facial droop, ptosis, hearing loss, otalgia, and tinnitus was likely caused by the lesion’s extension into the right eustachian tube orifice. It is interesting to note that patient’s past medical history of resolved Bell’s Palsy may have raised low clinical suspicion of NPC initially when evaluated in ED.

## Conclusions

This is the only case reported of NPC involving a sequence of symptoms associated with CN III, V, VI, VII, IX, XII that has been reported in the literature. Additionally, the involvement of CN VII makes this case particularly unique. Since this type of neoplasm does not warrant immediate surgical resection and there was a three-month difference between diagnosis and treatment, the mass continued to compress structures, leading to the progression of symptoms. With CN involvement being associated as a poor prognostic indicator for NPC disease progression, we concluded that thorough and frequent neurological examinations are warranted throughout the treatment course with NPC patients.

## References

[1] Chang ET, Ye W, Zeng YX, Adami HO (2021). The evolving epidemiology of nasopharyngeal carcinoma. Cancer Epidemiol Biomarkers Prev.

[2] Adham M, Lazim NM, Carlos R (2020). Clinical presentation of nasopharyngeal carcinoma. An evidence-based approach to the management of nasopharyngeal cancer.

[3] Hamoir M, Schmitz S, Suarez C (2018). The current role of salvage surgery in recurrent head and neck squamous cell carcinoma. Cancers (Basel).

[4] Christodoulopoulos N, Mastronikolis N, Tsiambas E (2019). Impact of different therapeutic regimens on survival of patients with nasopharyngeal carcinoma. JBUON.

[5] Bossi P, Chan AT, Licitra L (2021). Nasopharyngeal carcinoma: ESMO-EURACAN clinical practice guidelines for diagnosis, treatment and follow-up. Ann Oncol.

[6] Au KH, Ngan RK, Ng AW (2018). Treatment outcomes of nasopharyngeal carcinoma in modern era after intensity modulated radiotherapy (IMRT) in Hong Kong: a report of 3328 patients (HKNPCSG 1301 study). Oral Oncol.

[7] Huang CC, Fang FM, Chen HC, Hsu HC, Huang TL, Su YL, Chang YC (2017). Therapeutic outcome of nasopharyngeal carcinoma with cranial nerve palsy: a single institution experience of 104 patients. Onco Targets Ther.

[8] Beckmann YY, Deniz B, Gelal F, Seçil Y (2010). Third cranial nerve palsy as the presenting neuro-ophthalmic feature of nasopharyngeal carcinoma. J Neuroophthalmol.

[9] Low WK (2002). Facial palsy from metastatic nasopharyngeal carcinoma at various sites: three reports. Ear Nose Throat J.

[10] (2021). Neuroanatomy, cranial nerve 7 (facial). https://www.ncbi.nlm.nih.gov/books/NBK526119/..

